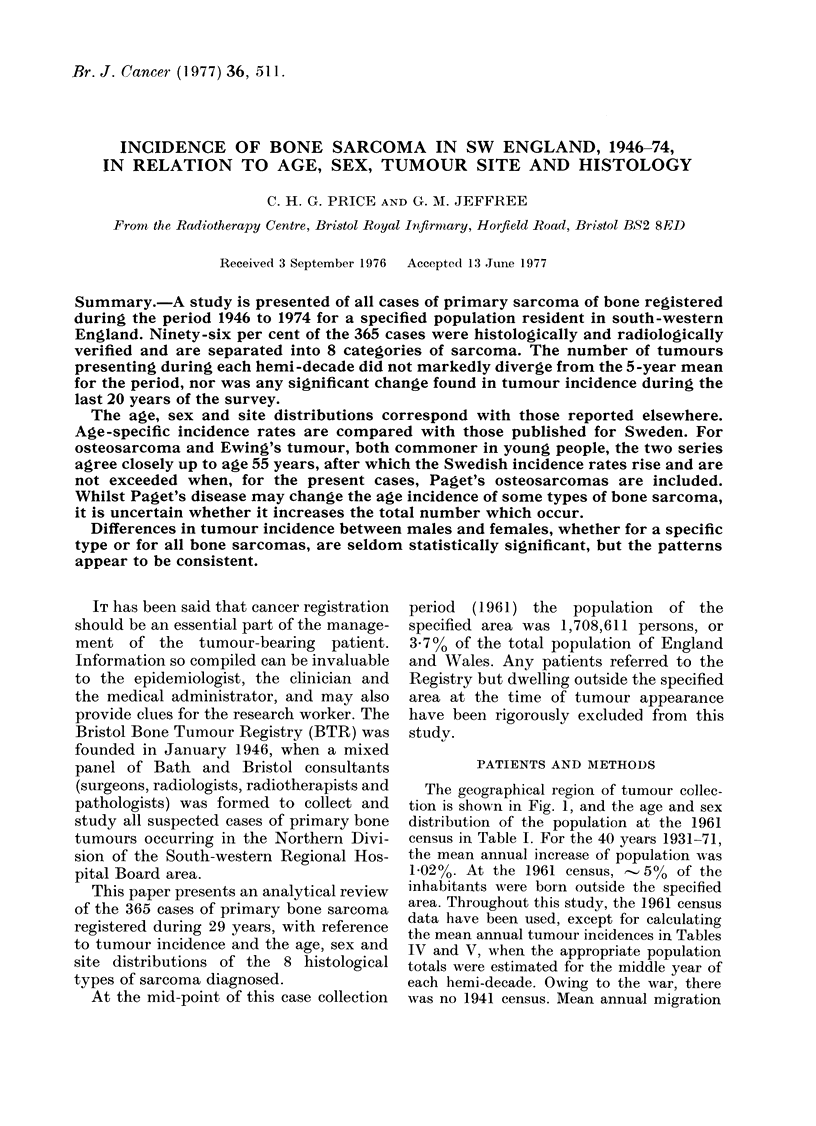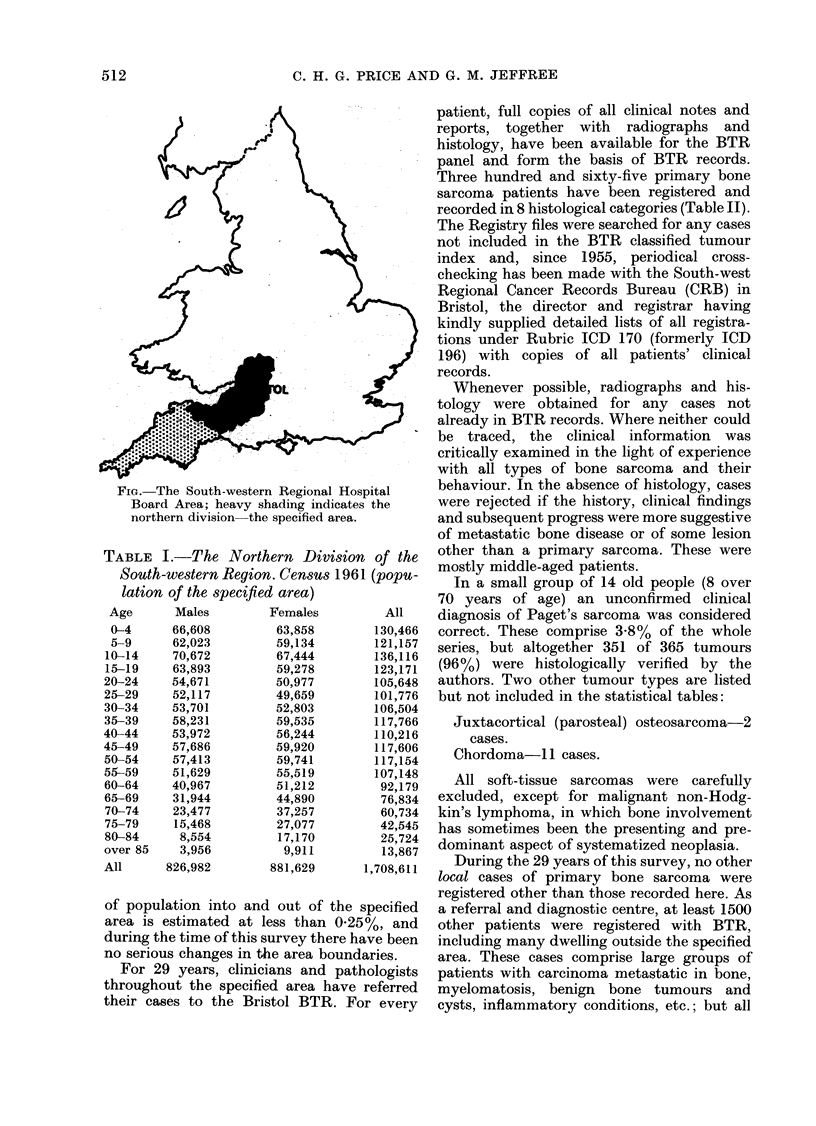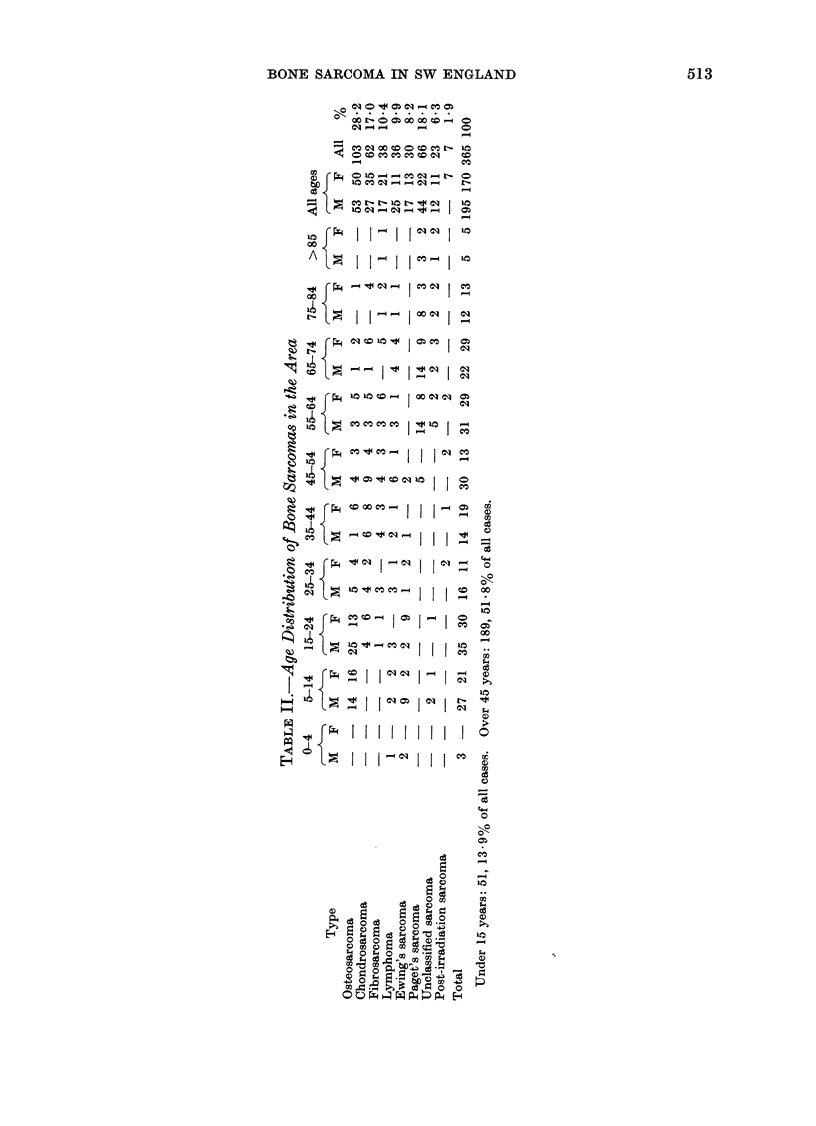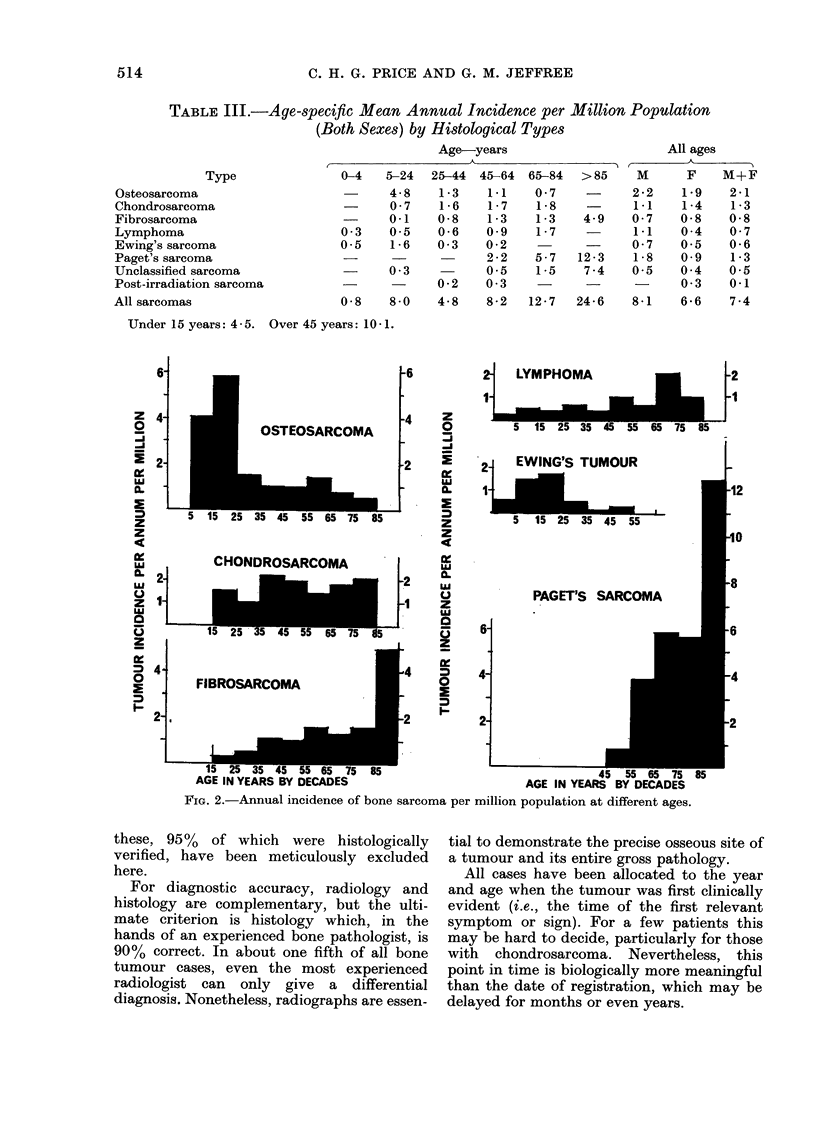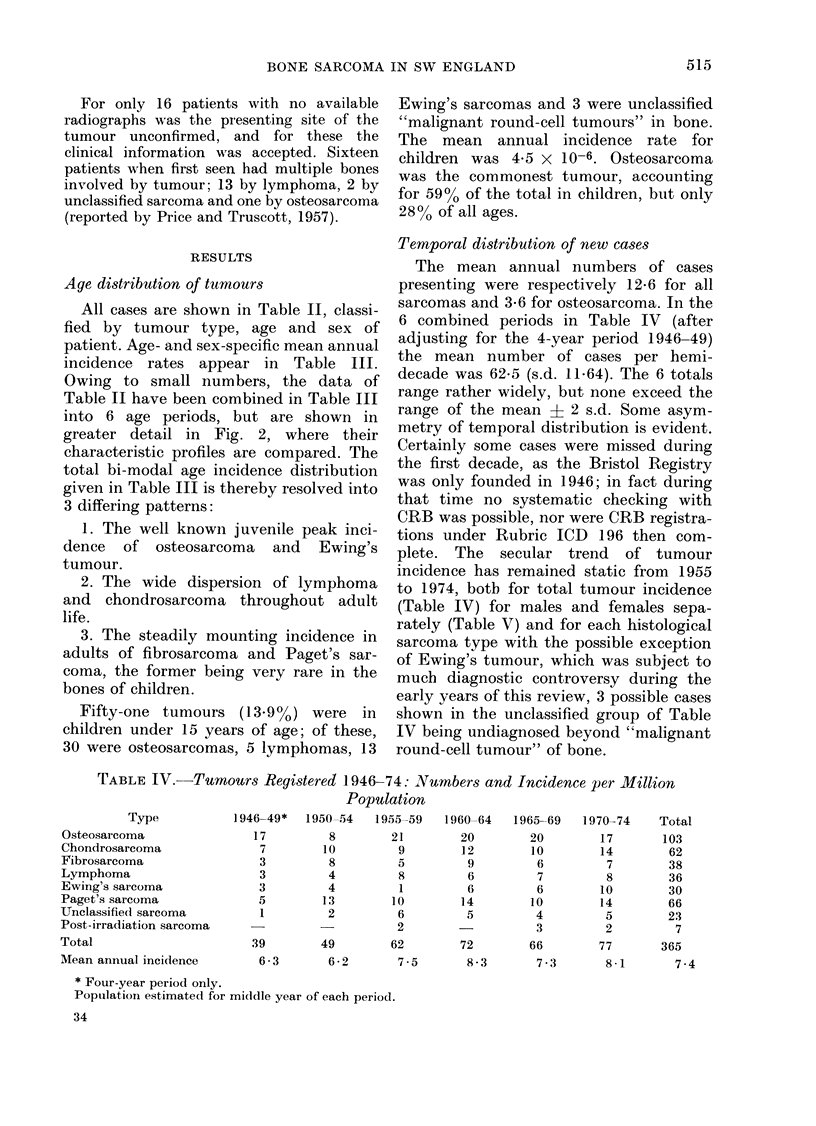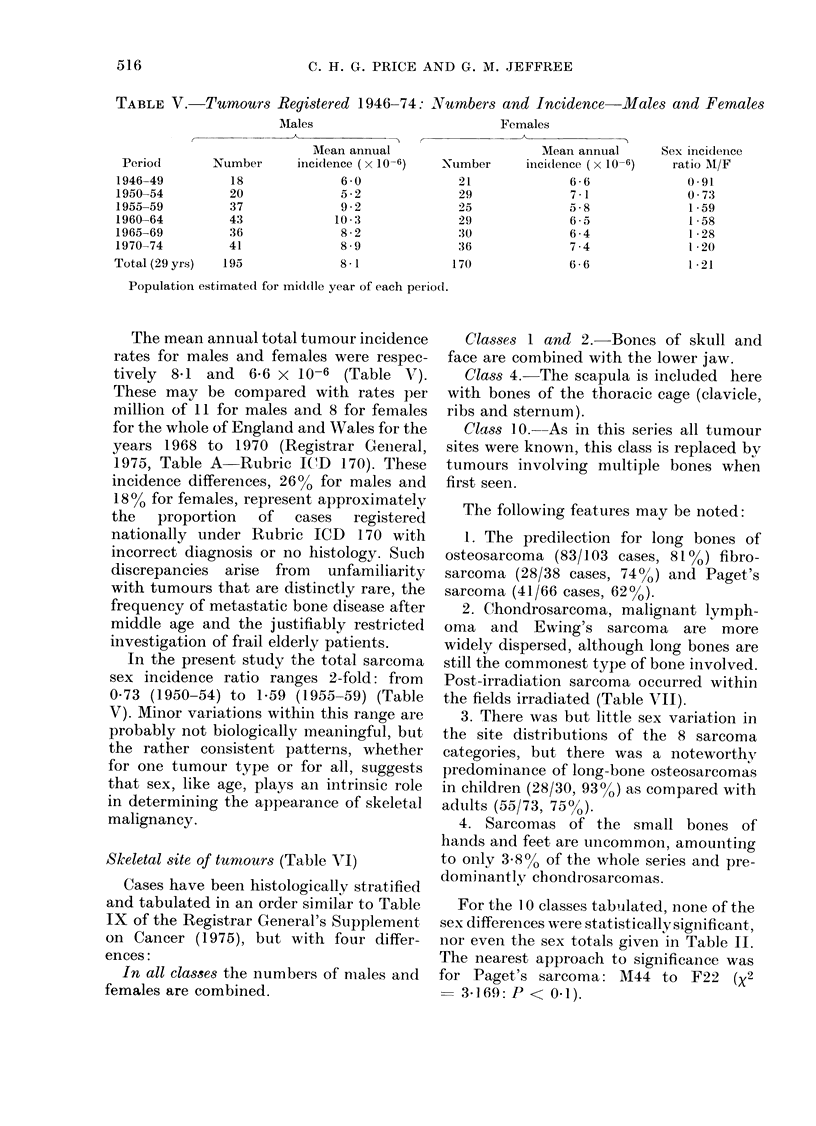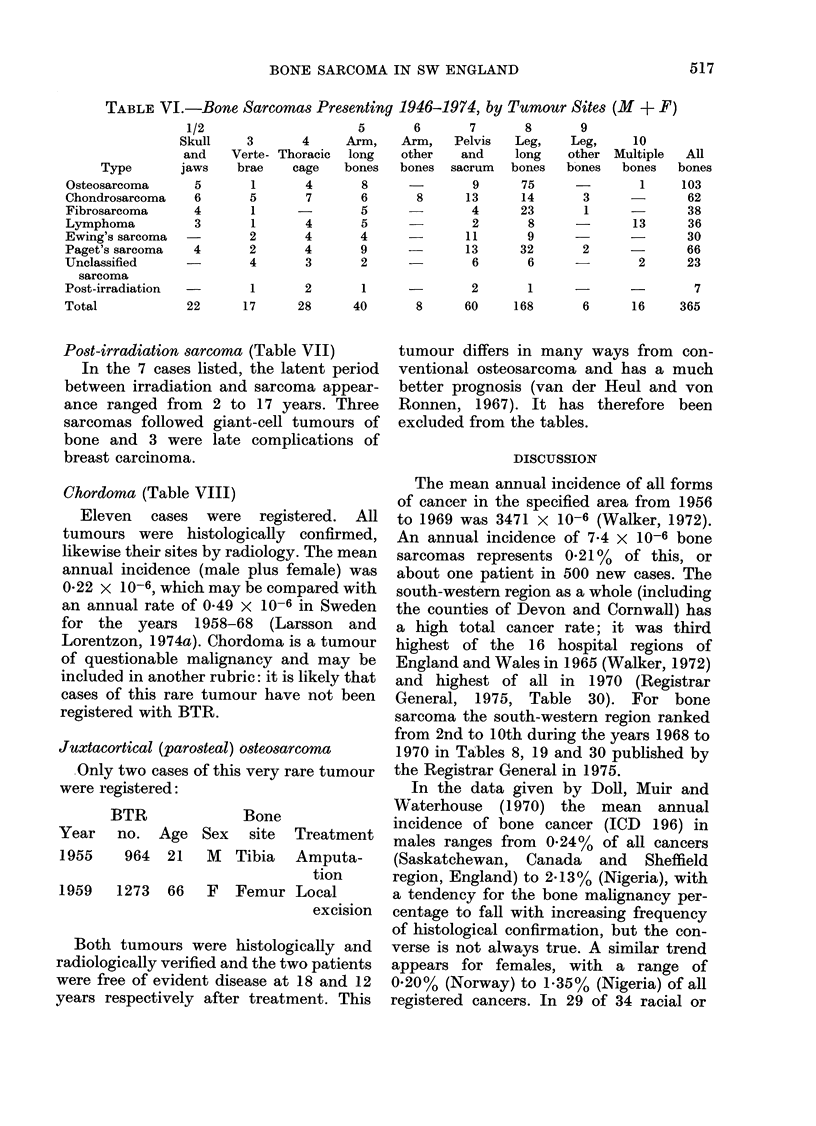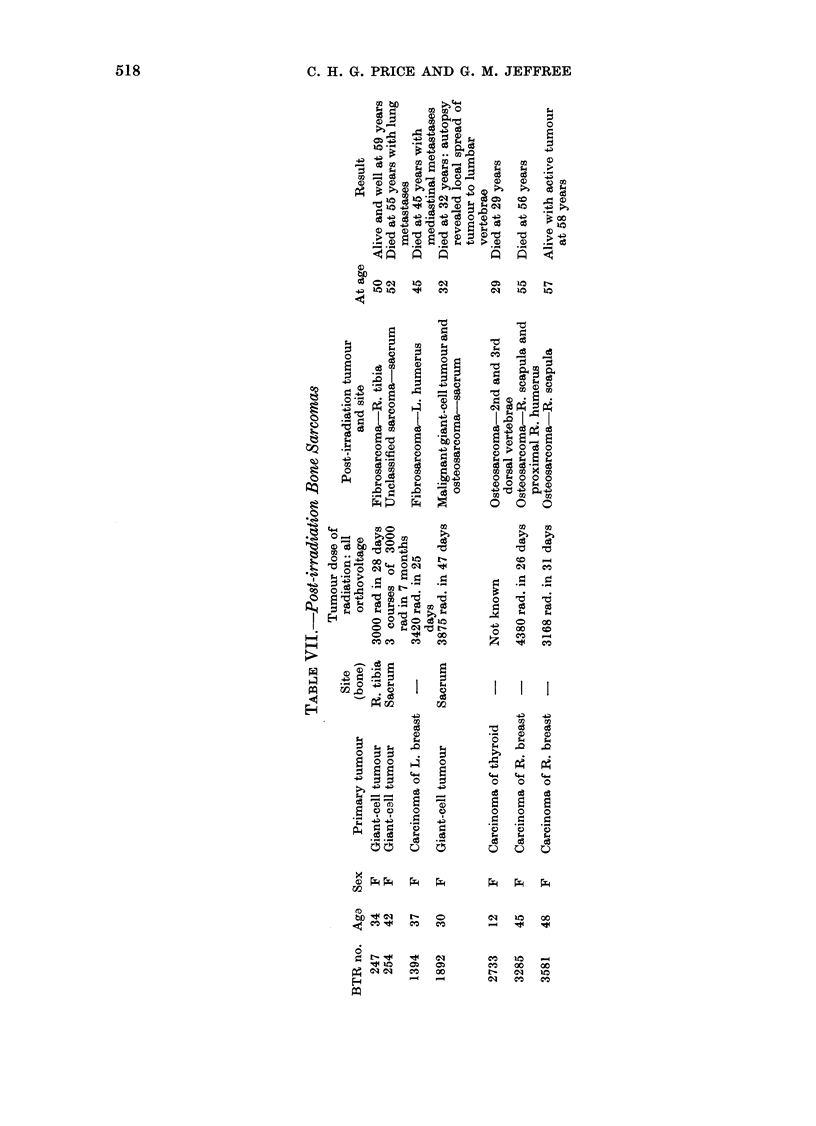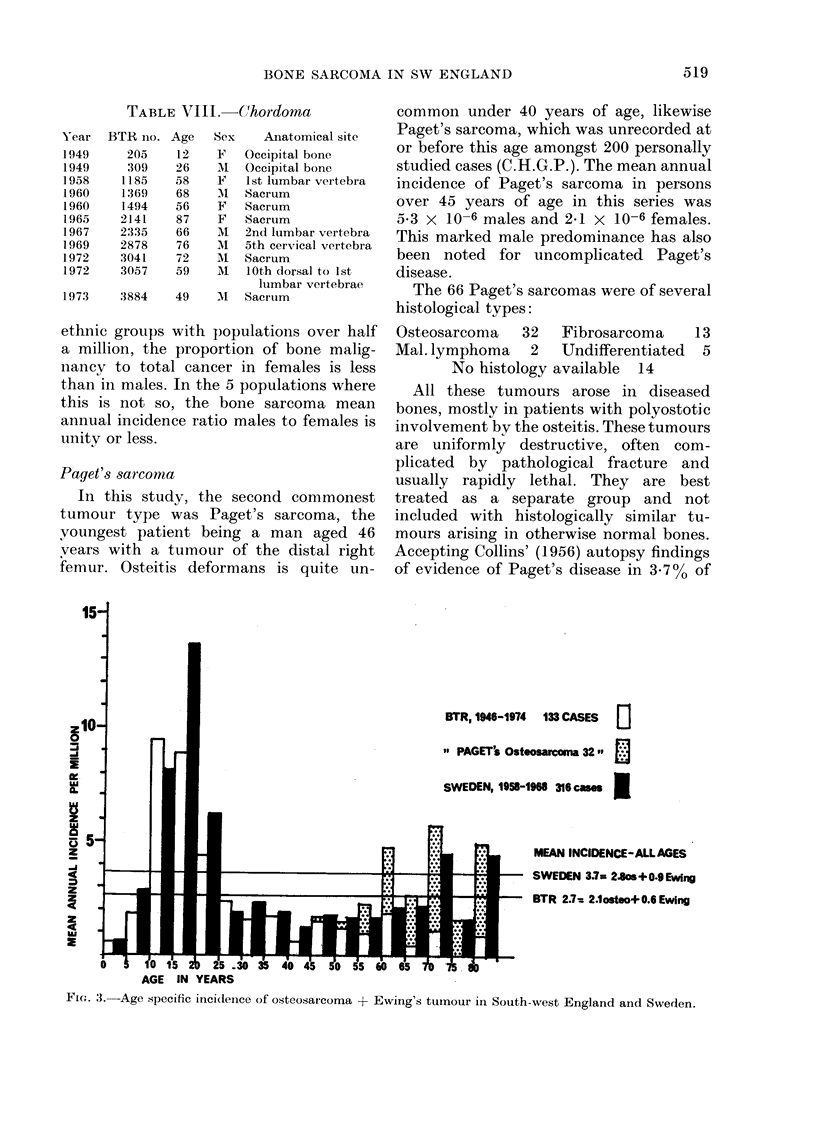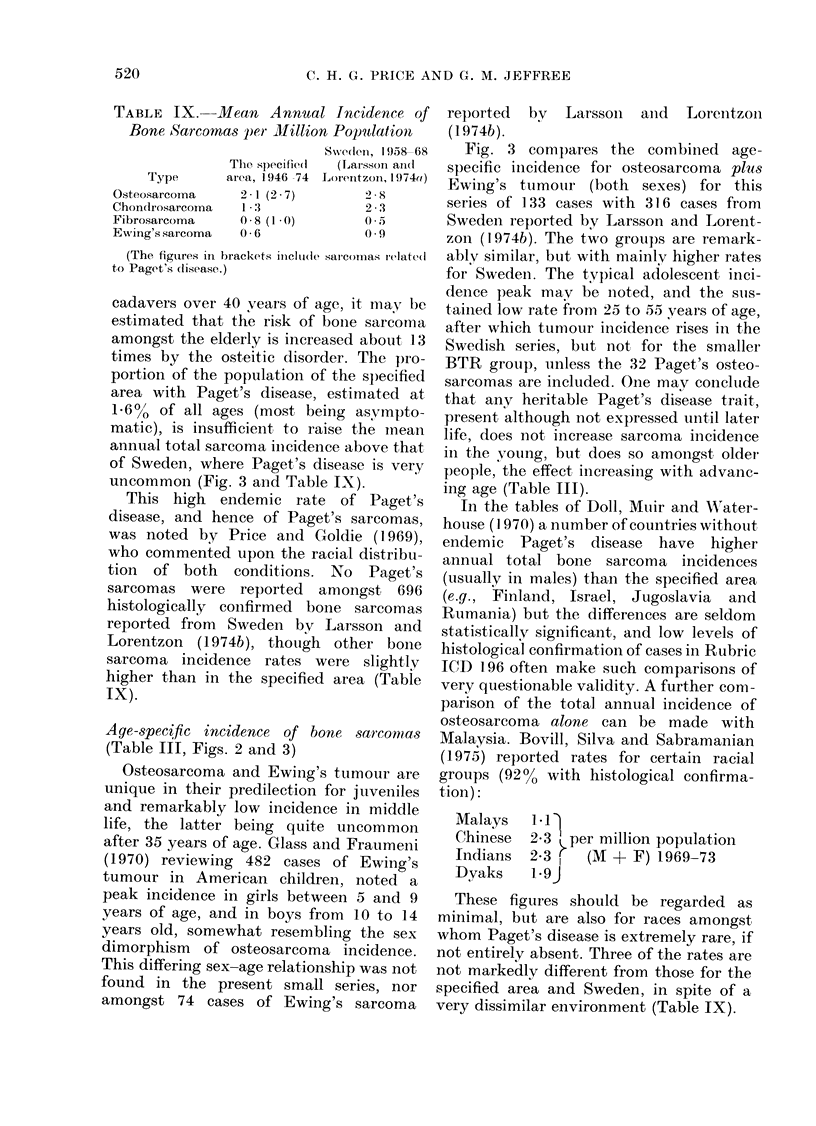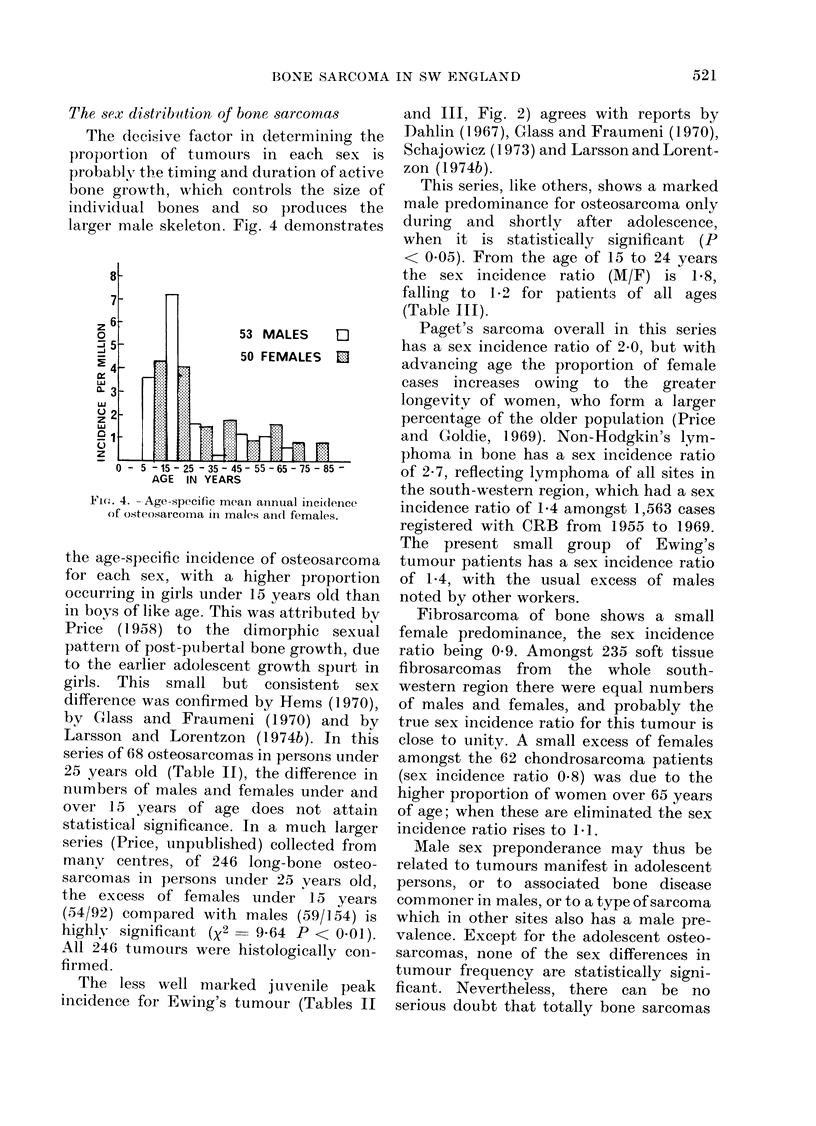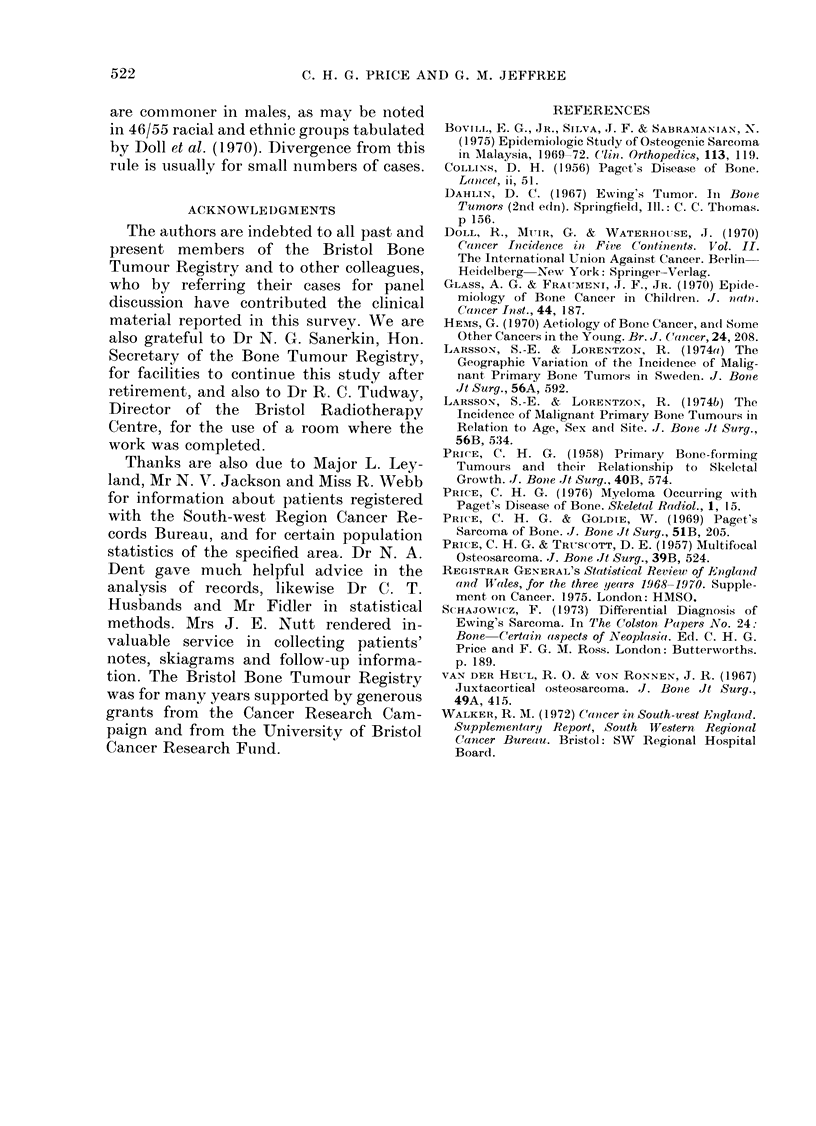# Incidence of bone sarcoma in SW England, 1946-74, in relation to age, sex, tumour site and histology.

**DOI:** 10.1038/bjc.1977.221

**Published:** 1977-10

**Authors:** C. H. Price, G. M. Jeffree

## Abstract

**Images:**


Br. J. Cancer (1977) 36, 511.

### INCIDENCE OF BONE SARCOMA IN SW ENGLAND, 1946–74, IN RELATION TO AGE, SEX, TUMOUR SITE AND HISTOLOGY

C. H. G. PRICE AND G. M. JEFFREE

*From the Radiotherapy Centre, Bristol Royal Infirmary, Horfield Road, Bristol BS2 8ED*

Received 3 September 1976 Accepted 13 June 1977

**Summary.—A study is presented of all cases of primary sarcoma of bone registered during the period 1946 to 1974 for a specified population resident in south-western England. Ninety-six per cent of the 365 cases were histologically and radiologically verified and are separated into 8 categories of sarcoma. The number of tumours presenting during each hemi-decade did not markedly diverge from the 5-year mean for the period, nor was any significant change found in tumour incidence during the last 20 years of the survey.**

**The age, sex and site distributions correspond with those reported elsewhere. Age-specific incidence rates are compared with those published for Sweden. For osteosarcoma and Ewing's tumour, both commoner in young people, the two series agree closely up to age 55 years, after which the Swedish incidence rates rise and are not exceeded when, for the present cases, Paget's osteosarcomas are included. Whilst Paget's disease may change the age incidence of some types of bone sarcoma, it is uncertain whether it increases the total number which occur.**

**Differences in tumour incidence between males and females, whether for a specific type or for all bone sarcomas, are seldom statistically significant, but the patterns appear to be consistent.**

It has been said that cancer registration should be an essential part of the management of the tumour-bearing patient. Information so compiled can be invaluable to the epidemiologist, the clinician and the medical administrator, and may also provide clues for the research worker. The Bristol Bone Tumour Registry (BTR) was founded in January 1946, when a mixed panel of Bath and Bristol consultants (surgeons, radiologists, radiotherapists and pathologists) was formed to collect and study all suspected cases of primary bone tumours occurring in the Northern Division of the South-western Regional Hospital Board area.

This paper presents an analytical review of the 365 cases of primary bone sarcoma registered during 29 years, with reference to tumour incidence and the age, sex and site distributions of the 8 histological types of sarcoma diagnosed.

At the mid-point of this case collection period (1961) the population of the specified area was 1,708,611 persons, or 3·7% of the total population of England and Wales. Any patients referred to the Registry but dwelling outside the specified area at the time of tumour appearance have been rigorously excluded from this study.

### PATIENTS AND METHODS

The geographical region of tumour collection is shown in Fig. 1, and the age and sex distribution of the population at the 1961 census in Table I. For the 40 years 1931–71, the mean annual increase of population was 1·02%. At the 1961 census, ~5% of the inhabitants were born outside the specified area. Throughout this study, the 1961 census data have been used, except for calculating the mean annual tumour incidences in Tables IV and V, when the appropriate population totals were estimated for the middle year of each hemi-decade. Owing to the war, there was no 1941 census. Mean annual migration

FIG.—The South-western Regional Hospital Board Area; heavy shading indicates the northern division—the specified area.

TABLE I.—*The Northern Division of the South-western Region. Census* 1961 (*population of the specified area*)

| Age | Males | Females | All |
|---|---|---|---|
| 0–4 | 66,608 | 63,858 | 130,466 |
| 5–9 | 62,023 | 59,134 | 121,157 |
| 10–14 | 70,672 | 67,444 | 136,116 |
| 15–19 | 63,893 | 59,278 | 123,171 |
| 20–24 | 54,671 | 50,977 | 105,648 |
| 25–29 | 52,117 | 49,659 | 101,776 |
| 30–34 | 53,701 | 52,803 | 106,504 |
| 35–39 | 58,231 | 59,535 | 117,766 |
| 40–44 | 53,972 | 56,244 | 110,216 |
| 45–49 | 57,686 | 59,920 | 117,606 |
| 50–54 | 57,413 | 59,741 | 117,154 |
| 55–59 | 51,629 | 55,519 | 107,148 |
| 60–64 | 40,967 | 51,212 | 92,179 |
| 65–69 | 31,944 | 44,890 | 76,834 |
| 70–74 | 23,477 | 37,257 | 60,734 |
| 75–79 | 15,468 | 27,077 | 42,545 |
| 80–84 | 8,554 | 17,170 | 25,724 |
| over 85 | 3,956 | 9,911 | 13,867 |
| All | 826,982 | 881,629 | 1,708,611 |

of population into and out of the specified area is estimated at less than 0·25%, and during the time of this survey there have been no serious changes in the area boundaries.

For 29 years, clinicians and pathologists throughout the specified area have referred their cases to the Bristol BTR. For every patient, full copies of all clinical notes and reports, together with radiographs and histology, have been available for the BTR panel and form the basis of BTR records. Three hundred and sixty-five primary bone sarcoma patients have been registered and recorded in 8 histological categories (Table II). The Registry files were searched for any cases not included in the BTR classified tumour index and, since 1955, periodical cross-checking has been made with the South-west Regional Cancer Records Bureau (CRB) in Bristol, the director and registrar having kindly supplied detailed lists of all registrations under Rubric ICD 170 (formerly ICD 196) with copies of all patients' clinical records.

Whenever possible, radiographs and histology were obtained for any cases not already in BTR records. Where neither could be traced, the clinical information was critically examined in the light of experience with all types of bone sarcoma and their behaviour. In the absence of histology, cases were rejected if the history, clinical findings and subsequent progress were more suggestive of metastatic bone disease or of some lesion other than a primary sarcoma. These were mostly middle-aged patients.

In a small group of 14 old people (8 over 70 years of age) an unconfirmed clinical diagnosis of Paget's sarcoma was considered correct. These comprise 3·8% of the whole series, but altogether 351 of 365 tumours (96%) were histologically verified by the authors. Two other tumour types are listed but not included in the statistical tables:

Juxtacortical (parosteal) osteosarcoma—2 cases.
Chordoma—11 cases.

All soft-tissue sarcomas were carefully excluded, except for malignant non-Hodgkin's lymphoma, in which bone involvement has sometimes been the presenting and predominant aspect of systematized neoplasia.

During the 29 years of this survey, no other *local* cases of primary bone sarcoma were registered other than those recorded here. As a referral and diagnostic centre, at least 1500 other patients were registered with BTR, including many dwelling outside the specified area. These cases comprise large groups of patients with carcinoma metastatic in bone, myelomatosis, benign bone tumours and cysts, inflammatory conditions, etc.; but all

TABLE II.—*Age Distribution of Bone Sarcomas in the Area*

| Type | 0–4 | | 5–14 | | 15–24 | | 25–34 | | 35–44 | | 45–54 | | 55–64 | | 65–74 | | 75–84 | | >85 | | All ages | | | |
|---|---|---|---|---|---|---|---|---|---|---|---|---|---|---|---|---|---|---|---|---|---|---|---|---|
| | M | F | M | F | M | F | M | F | M | F | M | F | M | F | M | F | M | F | M | F | M | F | All | % |
| Osteosarcoma | — | — | 14 | 16 | 25 | 13 | 5 | 4 | 1 | 6 | 4 | 3 | 3 | 5 | 1 | 2 | — | 1 | — | — | 53 | 50 | 103 | 28·2 |
| Chondrosarcoma | — | — | — | — | 4 | 6 | 4 | 2 | 6 | 8 | 9 | 4 | 3 | 5 | 1 | 6 | — | 4 | — | — | 27 | 35 | 62 | 17·0 |
| Fibrosarcoma | — | — | — | — | 1 | 1 | 3 | — | 4 | 3 | 4 | 3 | 3 | 6 | — | 5 | 1 | 2 | 1 | 1 | 17 | 21 | 38 | 10·4 |
| Lymphoma | 1 | — | 2 | 2 | 3 | — | 3 | 1 | 2 | 1 | 6 | 1 | 3 | 1 | 4 | 4 | 1 | 1 | — | — | 25 | 11 | 36 | 9·9 |
| Ewing's sarcoma | 2 | — | 9 | 2 | 2 | 9 | 1 | 2 | 1 | — | 2 | — | — | — | — | — | — | — | — | — | 17 | 13 | 30 | 8·2 |
| Paget's sarcoma | — | — | — | — | — | — | — | — | — | — | 5 | — | 14 | 8 | 14 | 9 | 8 | 3 | 3 | 2 | 44 | 22 | 66 | 18·1 |
| Unclassified sarcoma | — | — | 2 | 1 | — | 1 | — | — | — | — | — | — | 5 | 2 | 2 | 3 | 2 | 2 | 1 | 2 | 12 | 11 | 23 | 6·3 |
| Post-irradiation sarcoma | — | — | — | — | — | — | — | 2 | — | 1 | — | 2 | — | 2 | — | — | — | — | — | — | — | 7 | 7 | 1·9 |
| Total | 3 | — | 27 | 21 | 35 | 30 | 16 | 11 | 14 | 19 | 30 | 13 | 31 | 29 | 22 | 29 | 12 | 13 | 5 | 5 | 195 | 170 | 365 | 100 |

Under 15 years: 51, 13·9% of all cases. Over 45 years: 189, 51·8% of all cases.

TABLE III.—*Age-specific Mean Annual Incidence per Million Population (Both Sexes) by Histological Types*

| Type | Age—years | | | | | | All ages | | |
|---|---|---|---|---|---|---|---|---|---|
| | 0–4 | 5–24 | 25–44 | 45–64 | 65–84 | >85 | M | F | M+F |
| Osteosarcoma | — | 4·8 | 1·3 | 1·1 | 0·7 | — | 2·2 | 1·9 | 2·1 |
| Chondrosarcoma | — | 0·7 | 1·6 | 1·7 | 1·8 | — | 1·1 | 1·4 | 1·3 |
| Fibrosarcoma | — | 0·1 | 0·8 | 1·3 | 1·3 | 4·9 | 0·7 | 0·8 | 0·8 |
| Lymphoma | 0·3 | 0·5 | 0·6 | 0·9 | 1·7 | — | 1·1 | 0·4 | 0·7 |
| Ewing's sarcoma | 0·5 | 1·6 | 0·3 | 0·2 | — | — | 0·7 | 0·5 | 0·6 |
| Paget's sarcoma | — | — | — | 2·2 | 5·7 | 12·3 | 1·8 | 0·9 | 1·3 |
| Unclassified sarcoma | — | 0·3 | — | 0·5 | 1·5 | 7·4 | 0·5 | 0·4 | 0·5 |
| Post-irradiation sarcoma | — | — | 0·2 | 0·3 | — | — | — | 0·3 | 0·1 |
| All sarcomas | 0·8 | 8·0 | 4·8 | 8·2 | 12·7 | 24·6 | 8·1 | 6·6 | 7·4 |

Under 15 years: 4·5. Over 45 years: 10·1.

FIG. 2.—Annual incidence of bone sarcoma per million population at different ages.

these, 95% of which were histologically verified, have been meticulously excluded here.

For diagnostic accuracy, radiology and histology are complementary, but the ultimate criterion is histology which, in the hands of an experienced bone pathologist, is 90% correct. In about one fifth of all bone tumour cases, even the most experienced radiologist can only give a differential diagnosis. Nonetheless, radiographs are essential to demonstrate the precise osseous site of a tumour and its entire gross pathology.

All cases have been allocated to the year and age when the tumour was first clinically evident (*i.e.*, the time of the first relevant symptom or sign). For a few patients this may be hard to decide, particularly for those with chondrosarcoma. Nevertheless, this point in time is biologically more meaningful than the date of registration, which may be delayed for months or even years.

For only 16 patients with no available radiographs was the presenting site of the tumour unconfirmed, and for these the clinical information was accepted. Sixteen patients when first seen had multiple bones involved by tumour; 13 by lymphoma, 2 by unclassified sarcoma and one by osteosarcoma (reported by Price and Truscott, 1957).

### RESULTS

### *Age distribution of tumours*

All cases are shown in Table II, classified by tumour type, age and sex of patient. Age- and sex-specific mean annual incidence rates appear in Table III. Owing to small numbers, the data of Table II have been combined in Table III into 6 age periods, but are shown in greater detail in Fig. 2, where their characteristic profiles are compared. The total bi-modal age incidence distribution given in Table III is thereby resolved into 3 differing patterns:

1. The well known juvenile peak incidence of osteosarcoma and Ewing's tumour.
2. The wide dispersion of lymphoma and chondrosarcoma throughout adult life.
3. The steadily mounting incidence in adults of fibrosarcoma and Paget's sarcoma, the former being very rare in the bones of children.

Fifty-one tumours (13·9%) were in children under 15 years of age; of these, 30 were osteosarcomas, 5 lymphomas, 13 Ewing's sarcomas and 3 were unclassified "malignant round-cell tumours" in bone. The mean annual incidence rate for children was $4{\cdot}5 \times 10^{-6}$. Osteosarcoma was the commonest tumour, accounting for 59% of the total in children, but only 28% of all ages.

### *Temporal distribution of new cases*

The mean annual numbers of cases presenting were respectively 12·6 for all sarcomas and 3·6 for osteosarcoma. In the 6 combined periods in Table IV (after adjusting for the 4-year period 1946–49) the mean number of cases per hemi-decade was 62·5 (s.d. 11·64). The 6 totals range rather widely, but none exceed the range of the mean ± 2 s.d. Some asymmetry of temporal distribution is evident. Certainly some cases were missed during the first decade, as the Bristol Registry was only founded in 1946; in fact during that time no systematic checking with CRB was possible, nor were CRB registrations under Rubric ICD 196 then complete. The secular trend of tumour incidence has remained static from 1955 to 1974, both for total tumour incidence (Table IV) for males and females separately (Table V) and for each histological sarcoma type with the possible exception of Ewing's tumour, which was subject to much diagnostic controversy during the early years of this review, 3 possible cases shown in the unclassified group of Table IV being undiagnosed beyond "malignant round-cell tumour" of bone.

TABLE IV.—*Tumours Registered* 1946–74*: Numbers and Incidence per Million Population*

| Type | 1946–49* | 1950–54 | 1955–59 | 1960–64 | 1965–69 | 1970–74 | Total |
|---|---|---|---|---|---|---|---|
| Osteosarcoma | 17 | 8 | 21 | 20 | 20 | 17 | 103 |
| Chondrosarcoma | 7 | 10 | 9 | 12 | 10 | 14 | 62 |
| Fibrosarcoma | 3 | 8 | 5 | 9 | 6 | 7 | 38 |
| Lymphoma | 3 | 4 | 8 | 6 | 7 | 8 | 36 |
| Ewing's sarcoma | 3 | 4 | 1 | 6 | 6 | 10 | 30 |
| Paget's sarcoma | 5 | 13 | 10 | 14 | 10 | 14 | 66 |
| Unclassified sarcoma | 1 | 2 | 6 | 5 | 4 | 5 | 23 |
| Post-irradiation sarcoma | — | — | 2 | — | 3 | 2 | 7 |
| Total | 39 | 49 | 62 | 72 | 66 | 77 | 365 |
| Mean annual incidence | 6·3 | 6·2 | 7·5 | 8·3 | 7·3 | 8·1 | 7·4 |

* Four-year period only.
Population estimated for middle year of each period.

TABLE V.—*Tumours Registered* 1946–74: *Numbers and Incidence—Males and Females*

| | Males | | Females | | |
|---|---|---|---|---|---|
| Period | Number | Mean annual incidence ($\times 10^{-6}$) | Number | Mean annual incidence ($\times 10^{-6}$) | Sex incidence ratio M/F |
| 1946–49 | 18 | 6·0 | 21 | 6·6 | 0·91 |
| 1950–54 | 20 | 5·2 | 29 | 7·1 | 0·73 |
| 1955–59 | 37 | 9·2 | 25 | 5·8 | 1·59 |
| 1960–64 | 43 | 10·3 | 29 | 6·5 | 1·58 |
| 1965–69 | 36 | 8·2 | 30 | 6·4 | 1·28 |
| 1970–74 | 41 | 8·9 | 36 | 7·4 | 1·20 |
| Total (29 yrs) | 195 | 8·1 | 170 | 6·6 | 1·21 |

Population estimated for middle year of each period.

The mean annual total tumour incidence rates for males and females were respectively 8·1 and 6·6 $\times 10^{-6}$ (Table V). These may be compared with rates per million of 11 for males and 8 for females for the whole of England and Wales for the years 1968 to 1970 (Registrar General, 1975, Table A—Rubric ICD 170). These incidence differences, 26% for males and 18% for females, represent approximately the proportion of cases registered nationally under Rubric ICD 170 with incorrect diagnosis or no histology. Such discrepancies arise from unfamiliarity with tumours that are distinctly rare, the frequency of metastatic bone disease after middle age and the justifiably restricted investigation of frail elderly patients.

In the present study the total sarcoma sex incidence ratio ranges 2-fold: from 0·73 (1950–54) to 1·59 (1955–59) (Table V). Minor variations within this range are probably not biologically meaningful, but the rather consistent patterns, whether for one tumour type or for all, suggests that sex, like age, plays an intrinsic role in determining the appearance of skeletal malignancy.

*Skeletal site of tumours* (Table VI)

Cases have been histologically stratified and tabulated in an order similar to Table IX of the Registrar General's Supplement on Cancer (1975), but with four differences:

*In all classes* the numbers of males and females are combined.

*Classes* 1 *and* 2.—Bones of skull and face are combined with the lower jaw.

*Class* 4.—The scapula is included here with bones of the thoracic cage (clavicle, ribs and sternum).

*Class* 10.—As in this series all tumour sites were known, this class is replaced by tumours involving multiple bones when first seen.

The following features may be noted:

1. The predilection for long bones of osteosarcoma (83/103 cases, 81%) fibrosarcoma (28/38 cases, 74%) and Paget's sarcoma (41/66 cases, 62%).
2. Chondrosarcoma, malignant lymphoma and Ewing's sarcoma are more widely dispersed, although long bones are still the commonest type of bone involved. Post-irradiation sarcoma occurred within the fields irradiated (Table VII).
3. There was but little sex variation in the site distributions of the 8 sarcoma categories, but there was a noteworthy predominance of long-bone osteosarcomas in children (28/30, 93%) as compared with adults (55/73, 75%).
4. Sarcomas of the small bones of hands and feet are uncommon, amounting to only 3·8% of the whole series and predominantly chondrosarcomas.

For the 10 classes tabulated, none of the sex differences were statistically significant, nor even the sex totals given in Table II. The nearest approach to significance was for Paget's sarcoma: M44 to F22 ($\chi^2 = 3{\cdot}169$: $P < 0{\cdot}1$).

TABLE VI.—*Bone Sarcomas Presenting 1946–1974, by Tumour Sites (M + F)*

| Type | 1/2 Skull and jaws | 3 Verte-brae | 4 Thoracic cage | 5 Arm, long bones | 6 Arm, other bones | 7 Pelvis and sacrum | 8 Leg, long bones | 9 Leg, other bones | 10 Multiple bones | All bones |
|---|---|---|---|---|---|---|---|---|---|---|
| Osteosarcoma | 5 | 1 | 4 | 8 | — | 9 | 75 | — | 1 | 103 |
| Chondrosarcoma | 6 | 5 | 7 | 6 | 8 | 13 | 14 | 3 | — | 62 |
| Fibrosarcoma | 4 | 1 | — | 5 | — | 4 | 23 | 1 | — | 38 |
| Lymphoma | 3 | 1 | 4 | 5 | — | 2 | 8 | — | 13 | 36 |
| Ewing's sarcoma | — | 2 | 4 | 4 | — | 11 | 9 | — | — | 30 |
| Paget's sarcoma | 4 | 2 | 4 | 9 | — | 13 | 32 | 2 | — | 66 |
| Unclassified sarcoma | — | 4 | 3 | 2 | — | 6 | 6 | — | 2 | 23 |
| Post-irradiation | — | 1 | 2 | 1 | — | 2 | 1 | — | — | 7 |
| Total | 22 | 17 | 28 | 40 | 8 | 60 | 168 | 6 | 16 | 365 |

*Post-irradiation sarcoma* (Table VII)

In the 7 cases listed, the latent period between irradiation and sarcoma appearance ranged from 2 to 17 years. Three sarcomas followed giant-cell tumours of bone and 3 were late complications of breast carcinoma.

*Chordoma* (Table VIII)

Eleven cases were registered. All tumours were histologically confirmed, likewise their sites by radiology. The mean annual incidence (male plus female) was $0{\cdot}22 \times 10^{-6}$, which may be compared with an annual rate of $0{\cdot}49 \times 10^{-6}$ in Sweden for the years 1958–68 (Larsson and Lorentzon, 1974*a*). Chordoma is a tumour of questionable malignancy and may be included in another rubric: it is likely that cases of this rare tumour have not been registered with BTR.

*Juxtacortical (parosteal) osteosarcoma*

Only two cases of this very rare tumour were registered:

| Year | BTR no. | Age | Sex | Bone site | Treatment |
|---|---|---|---|---|---|
| 1955 | 964 | 21 | M | Tibia | Amputation |
| 1959 | 1273 | 66 | F | Femur | Local excision |

Both tumours were histologically and radiologically verified and the two patients were free of evident disease at 18 and 12 years respectively after treatment. This tumour differs in many ways from conventional osteosarcoma and has a much better prognosis (van der Heul and von Ronnen, 1967). It has therefore been excluded from the tables.

### DISCUSSION

The mean annual incidence of all forms of cancer in the specified area from 1956 to 1969 was $3471 \times 10^{-6}$ (Walker, 1972). An annual incidence of $7{\cdot}4 \times 10^{-6}$ bone sarcomas represents 0·21% of this, or about one patient in 500 new cases. The south-western region as a whole (including the counties of Devon and Cornwall) has a high total cancer rate; it was third highest of the 16 hospital regions of England and Wales in 1965 (Walker, 1972) and highest of all in 1970 (Registrar General, 1975, Table 30). For bone sarcoma the south-western region ranked from 2nd to 10th during the years 1968 to 1970 in Tables 8, 19 and 30 published by the Registrar General in 1975.

In the data given by Doll, Muir and Waterhouse (1970) the mean annual incidence of bone cancer (ICD 196) in males ranges from 0·24% of all cancers (Saskatchewan, Canada and Sheffield region, England) to 2·13% (Nigeria), with a tendency for the bone malignancy percentage to fall with increasing frequency of histological confirmation, but the converse is not always true. A similar trend appears for females, with a range of 0·20% (Norway) to 1·35% (Nigeria) of all registered cancers. In 29 of 34 racial or

TABLE VII.—*Post-irradiation Bone Sarcomas*

| BTR no. | Age | Sex | Primary tumour | Site (bone) | Tumour dose of radiation: all orthovoltage | Post-irradiation tumour and site | At age | Result |
|---|---|---|---|---|---|---|---|---|
| 247 | 34 | F | Giant-cell tumour | R. tibia | 3000 rad in 28 days | Fibrosarcoma—R. tibia | 50 | Alive and well at 59 years |
| 254 | 42 | F | Giant-cell tumour | Sacrum | 3 courses of 3000 rad in 7 months | Unclassified sarcoma—sacrum | 52 | Died at 55 years with lung metastases |
| 1394 | 37 | F | Carcinoma of L. breast | — | 3420 rad. in 25 days | Fibrosarcoma—L. humerus | 45 | Died at 45 years with mediastinal metastases |
| 1892 | 30 | F | Giant-cell tumour | Sacrum | 3875 rad. in 47 days | Malignant giant-cell tumour and osteosarcoma—sacrum | 32 | Died at 32 years: autopsy revealed local spread of tumour to lumbar vertebrae |
| 2733 | 12 | F | Carcinoma of thyroid | — | Not known | Osteosarcoma—2nd and 3rd dorsal vertebrae | 29 | Died at 29 years |
| 3285 | 45 | F | Carcinoma of R. breast | — | 4380 rad. in 26 days | Osteosarcoma—R. scapula and proximal R. humerus | 55 | Died at 56 years |
| 3581 | 48 | F | Carcinoma of R. breast | — | 3168 rad. in 31 days | Osteosarcoma—R. scapula | 57 | Alive with active tumour at 58 years |

TABLE VIII.—*Chordoma*

| Year | BTR no. | Age | Sex | Anatomical site |
|---|---|---|---|---|
| 1949 | 205 | 12 | F | Occipital bone |
| 1949 | 309 | 26 | M | Occipital bone |
| 1958 | 1185 | 58 | F | 1st lumbar vertebra |
| 1960 | 1369 | 68 | M | Sacrum |
| 1960 | 1494 | 56 | F | Sacrum |
| 1965 | 2141 | 87 | F | Sacrum |
| 1967 | 2335 | 66 | M | 2nd lumbar vertebra |
| 1969 | 2878 | 76 | M | 5th cervical vertebra |
| 1972 | 3041 | 72 | M | Sacrum |
| 1972 | 3057 | 59 | M | 10th dorsal to 1st lumbar vertebrae |
| 1973 | 3884 | 49 | M | Sacrum |

ethnic groups with populations over half a million, the proportion of bone malignancy to total cancer in females is less than in males. In the 5 populations where this is not so, the bone sarcoma mean annual incidence ratio males to females is unity or less.

*Paget's sarcoma*

In this study, the second commonest tumour type was Paget's sarcoma, the youngest patient being a man aged 46 years with a tumour of the distal right femur. Osteitis deformans is quite uncommon under 40 years of age, likewise Paget's sarcoma, which was unrecorded at or before this age amongst 200 personally studied cases (C.H.G.P.). The mean annual incidence of Paget's sarcoma in persons over 45 years of age in this series was $5{\cdot}3 \times 10^{-6}$ males and $2{\cdot}1 \times 10^{-6}$ females. This marked male predominance has also been noted for uncomplicated Paget's disease.

The 66 Paget's sarcomas were of several histological types:

| | | | |
|---|---|---|---|
| Osteosarcoma | 32 | Fibrosarcoma | 13 |
| Mal. lymphoma | 2 | Undifferentiated | 5 |
| No histology available | 14 | | |

All these tumours arose in diseased bones, mostly in patients with polyostotic involvement by the osteitis. These tumours are uniformly destructive, often complicated by pathological fracture and usually rapidly lethal. They are best treated as a separate group and not included with histologically similar tumours arising in otherwise normal bones. Accepting Collins' (1956) autopsy findings of evidence of Paget's disease in 3·7% of

FIG. 3.—Age specific incidence of osteosarcoma + Ewing's tumour in South-west England and Sweden.

TABLE IX.—*Mean Annual Incidence of Bone Sarcomas per Million Population*

| Type | The specified area, 1946–74 | Sweden, 1958–68 (Larsson and Lorentzon, 1974*a*) |
|---|---|---|
| Osteosarcoma | 2·1 (2·7) | 2·8 |
| Chondrosarcoma | 1·3 | 2·3 |
| Fibrosarcoma | 0·8 (1·0) | 0·5 |
| Ewing's sarcoma | 0·6 | 0·9 |

(The figures in brackets include sarcomas related to Paget's disease.)

cadavers over 40 years of age, it may be estimated that the risk of bone sarcoma amongst the elderly is increased about 13 times by the osteitic disorder. The proportion of the population of the specified area with Paget's disease, estimated at 1·6% of all ages (most being asymptomatic), is insufficient to raise the mean annual total sarcoma incidence above that of Sweden, where Paget's disease is very uncommon (Fig. 3 and Table IX).

This high endemic rate of Paget's disease, and hence of Paget's sarcomas, was noted by Price and Goldie (1969), who commented upon the racial distribution of both conditions. No Paget's sarcomas were reported amongst 696 histologically confirmed bone sarcomas reported from Sweden by Larsson and Lorentzon (1974*b*), though other bone sarcoma incidence rates were slightly higher than in the specified area (Table IX).

*Age-specific incidence of bone sarcomas* (Table III, Figs. 2 and 3)

Osteosarcoma and Ewing's tumour are unique in their predilection for juveniles and remarkably low incidence in middle life, the latter being quite uncommon after 35 years of age. Glass and Fraumeni (1970) reviewing 482 cases of Ewing's tumour in American children, noted a peak incidence in girls between 5 and 9 years of age, and in boys from 10 to 14 years old, somewhat resembling the sex dimorphism of osteosarcoma incidence. This differing sex–age relationship was not found in the present small series, nor amongst 74 cases of Ewing's sarcoma reported by Larsson and Lorentzon (1974*b*).

Fig. 3 compares the combined age-specific incidence for osteosarcoma *plus* Ewing's tumour (both sexes) for this series of 133 cases with 316 cases from Sweden reported by Larsson and Lorentzon (1974*b*). The two groups are remarkably similar, but with mainly higher rates for Sweden. The typical adolescent incidence peak may be noted, and the sustained low rate from 25 to 55 years of age, after which tumour incidence rises in the Swedish series, but not for the smaller BTR group, unless the 32 Paget's osteosarcomas are included. One may conclude that any heritable Paget's disease trait, present although not expressed until later life, does not increase sarcoma incidence in the young, but does so amongst older people, the effect increasing with advancing age (Table III).

In the tables of Doll, Muir and Waterhouse (1970) a number of countries without endemic Paget's disease have higher annual total bone sarcoma incidences (usually in males) than the specified area (*e.g.*, Finland, Israel, Jugoslavia and Rumania) but the differences are seldom statistically significant, and low levels of histological confirmation of cases in Rubric ICD 196 often make such comparisons of very questionable validity. A further comparison of the total annual incidence of osteosarcoma *alone* can be made with Malaysia. Bovill, Silva and Sabramanian (1975) reported rates for certain racial groups (92% with histological confirmation):

| | | |
|---|---|---|
| Malays | 1·1 | per million population (M + F) 1969–73 |
| Chinese | 2·3 | |
| Indians | 2·3 | |
| Dyaks | 1·9 | |

These figures should be regarded as minimal, but are also for races amongst whom Paget's disease is extremely rare, if not entirely absent. Three of the rates are not markedly different from those for the specified area and Sweden, in spite of a very dissimilar environment (Table IX).

*The sex distribution of bone sarcomas*

The decisive factor in determining the proportion of tumours in each sex is probably the timing and duration of active bone growth, which controls the size of individual bones and so produces the larger male skeleton. Fig. 4 demonstrates the age-specific incidence of osteosarcoma for each sex, with a higher proportion occurring in girls under 15 years old than in boys of like age. This was attributed by Price (1958) to the dimorphic sexual pattern of post-pubertal bone growth, due to the earlier adolescent growth spurt in girls. This small but consistent sex difference was confirmed by Hems (1970), by Glass and Fraumeni (1970) and by Larsson and Lorentzon (1974*b*). In this series of 68 osteosarcomas in persons under 25 years old (Table II), the difference in numbers of males and females under and over 15 years of age does not attain statistical significance. In a much larger series (Price, unpublished) collected from many centres, of 246 long-bone osteosarcomas in persons under 25 years old, the excess of females under 15 years (54/92) compared with males (59/154) is highly significant ($\chi^2 = 9 \cdot 64$ $P < 0 \cdot 01$). All 246 tumours were histologically confirmed.

FIG. 4.—Age-specific mean annual incidence of osteosarcoma in males and females.

The less well marked juvenile peak incidence for Ewing's tumour (Tables II and III, Fig. 2) agrees with reports by Dahlin (1967), Glass and Fraumeni (1970), Schajowicz (1973) and Larsson and Lorentzon (1974*b*).

This series, like others, shows a marked male predominance for osteosarcoma only during and shortly after adolescence, when it is statistically significant ($P < 0 \cdot 05$). From the age of 15 to 24 years the sex incidence ratio (M/F) is 1·8, falling to 1·2 for patients of all ages (Table III).

Paget's sarcoma overall in this series has a sex incidence ratio of 2·0, but with advancing age the proportion of female cases increases owing to the greater longevity of women, who form a larger percentage of the older population (Price and Goldie, 1969). Non-Hodgkin's lymphoma in bone has a sex incidence ratio of 2·7, reflecting lymphoma of all sites in the south-western region, which had a sex incidence ratio of 1·4 amongst 1,563 cases registered with CRB from 1955 to 1969. The present small group of Ewing's tumour patients has a sex incidence ratio of 1·4, with the usual excess of males noted by other workers.

Fibrosarcoma of bone shows a small female predominance, the sex incidence ratio being 0·9. Amongst 235 soft tissue fibrosarcomas from the whole south-western region there were equal numbers of males and females, and probably the true sex incidence ratio for this tumour is close to unity. A small excess of females amongst the 62 chondrosarcoma patients (sex incidence ratio 0·8) was due to the higher proportion of women over 65 years of age; when these are eliminated the sex incidence ratio rises to 1·1.

Male sex preponderance may thus be related to tumours manifest in adolescent persons, or to associated bone disease commoner in males, or to a type of sarcoma which in other sites also has a male prevalence. Except for the adolescent osteosarcomas, none of the sex differences in tumour frequency are statistically significant. Nevertheless, there can be no serious doubt that totally bone sarcomas are commoner in males, as may be noted in 46/55 racial and ethnic groups tabulated by Doll *et al.* (1970). Divergence from this rule is usually for small numbers of cases.

ACKNOWLEDGMENTS

The authors are indebted to all past and present members of the Bristol Bone Tumour Registry and to other colleagues, who by referring their cases for panel discussion have contributed the clinical material reported in this survey. We are also grateful to Dr N. G. Sanerkin, Hon. Secretary of the Bone Tumour Registry, for facilities to continue this study after retirement, and also to Dr R. C. Tudway, Director of the Bristol Radiotherapy Centre, for the use of a room where the work was completed.

Thanks are also due to Major L. Leyland, Mr N. V. Jackson and Miss R. Webb for information about patients registered with the South-west Region Cancer Records Bureau, and for certain population statistics of the specified area. Dr N. A. Dent gave much helpful advice in the analysis of records, likewise Dr C. T. Husbands and Mr Fidler in statistical methods. Mrs J. E. Nutt rendered invaluable service in collecting patients' notes, skiagrams and follow-up information. The Bristol Bone Tumour Registry was for many years supported by generous grants from the Cancer Research Campaign and from the University of Bristol Cancer Research Fund.

REFERENCES

Bovill, E. G., Jr., Silva, J. F. & Sabramanian, N. (1975) Epidemiologic Study of Osteogenic Sarcoma in Malaysia, 1969–72. *Clin. Orthopedics*, **113**, 119.

Collins, D. H. (1956) Paget's Disease of Bone. *Lancet*, ii, 51.

Dahlin, D. C. (1967) Ewing's Tumor. In *Bone Tumors* (2nd edn). Springfield, Ill.: C. C. Thomas. p 156.

Doll, R., Muir, G. & Waterhouse, J. (1970) *Cancer Incidence in Five Continents. Vol. II.* The International Union Against Cancer. Berlin—Heidelberg—New York: Springer-Verlag.

Glass, A. G. & Fraumeni, J. F., Jr. (1970) Epidemiology of Bone Cancer in Children. *J. natn. Cancer Inst.*, **44**, 187.

Hems, G. (1970) Aetiology of Bone Cancer, and Some Other Cancers in the Young. *Br. J. Cancer*, **24**, 208.

Larsson, S.-E. & Lorentzon, R. (1974*a*) The Geographic Variation of the Incidence of Malignant Primary Bone Tumors in Sweden. *J. Bone Jt Surg.*, **56**A, 592.

Larsson, S.-E. & Lorentzon, R. (1974*b*) The Incidence of Malignant Primary Bone Tumours in Relation to Age, Sex and Site. *J. Bone Jt Surg.*, **56**B, 534.

Price, C. H. G. (1958) Primary Bone-forming Tumours and their Relationship to Skeletal Growth. *J. Bone Jt Surg.*, **40**B, 574.

Price, C. H. G. (1976) Myeloma Occurring with Paget's Disease of Bone. *Skeletal Radiol.*, **1**, 15.

Price, C. H. G. & Goldie, W. (1969) Paget's Sarcoma of Bone. *J. Bone Jt Surg.*, **51**B, 205.

Price, C. H. G. & Truscott, D. E. (1957) Multifocal Osteosarcoma. *J. Bone Jt Surg.*, **39**B, 524.

Registrar General's *Statistical Review of England and Wales, for the three years 1968–1970.* Supplement on Cancer. 1975. London: HMSO.

Schajowicz, F. (1973) Differential Diagnosis of Ewing's Sarcoma. In *The Colston Papers No. 24: Bone—Certain aspects of Neoplasia.* Ed. C. H. G. Price and F. G. M. Ross. London: Butterworths. p. 189.

van der Heul, R. O. & von Ronnen, J. R. (1967) Juxtacortical osteosarcoma. *J. Bone Jt Surg.*, **49**A, 415.

Walker, R. M. (1972) *Cancer in South-west England. Supplementary Report, South Western Regional Cancer Bureau.* Bristol: SW Regional Hospital Board.